# Supplementary material for: The tumor microenvironment shows a hierarchy of cell-cell interactions dominated by fibroblasts
Source: Nat Commun. 2023 Sep 19;14:5810. doi: 10.1038/s41467-023-41518-w (PMC10509226; doi:10.1038/s41467-023-41518-w)
Supplement: Supplementary file 1 — Supplementary Information [file 41467_2023_41518_MOESM1_ESM.pdf]

The tumor microenvironment shows a hierarchy of cell-cell interactions dominated by fibroblasts

Supplementary Figures 1-7 and Supplementary Tables 1-3

Mayer and Milo et al.

Supplementary Figure 1

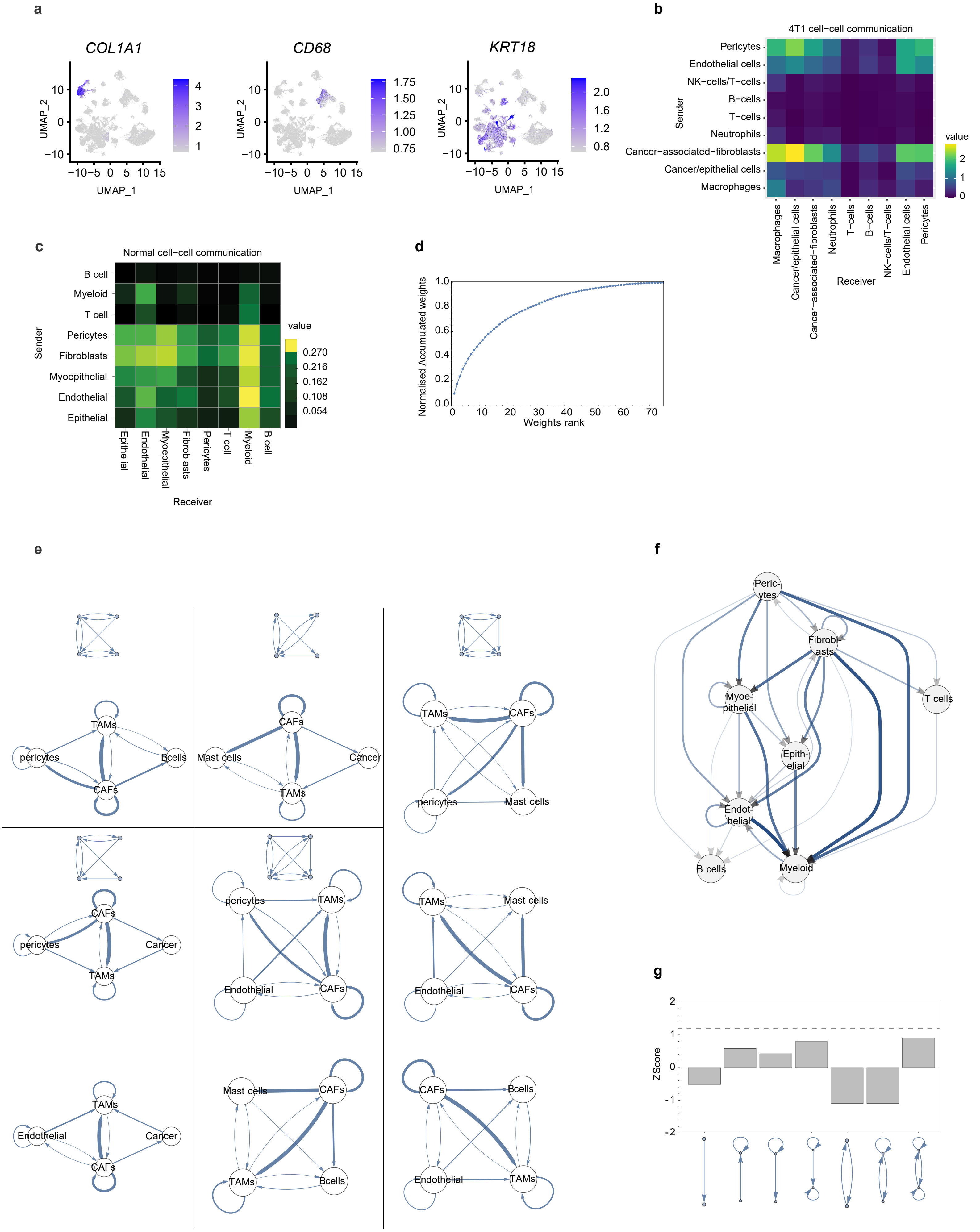

Supplementary Figure 1. The CAF-TAM subgraph is a dominant TME interaction. (**a**) UMAP visualization shows the main markers for the human scRNA-seq [1] clusters of fibroblasts (COL1A1), macrophages (CD68), and cancer (KRT18). (**b**) Heatmap of interaction strengths between pairs of cells based on cumulative ligand-receptor interaction scores using CellChat [2] applied to mouse breast cancer scRNA-seq data [3]. (**c**) Heatmap of interaction strengths between pairs of cells based on cumulative ligand-receptor interaction scores using CellChat applied to normal breast tissue samples[1]. Source data for (b) and (c) are provided as a Source Data file. (**d**) The contribution of weight ranks to the total weights in the human breast TME network. 50% of the interactions contribute to 90% of the weights. (**e**) Four-cell circuits with different network motifs in the human breast TME network. (**f**) Illustration of the structure of the normal breast tissue network based on the analysis in (**c**) shows hierarchy. A weighted directed graph was generated from the interaction weights matrix. The network was pruned down by considering only the edges with the strongest 50% of weights. The network was then plotted using Layered Digraph Embedding to highlight the inherent hierarchy (the root node was chosen as the node with the highest weighted outdegree). (**g**) No statistically significant motifs were revealed by network motif analysis of 2-node circuits of the normal breast tissue network (see Methods). The dashed line represents a 0.05 p-value threshold. Same source data as for (c).

Mayer and Milo et al.

Supplementary Figure 2

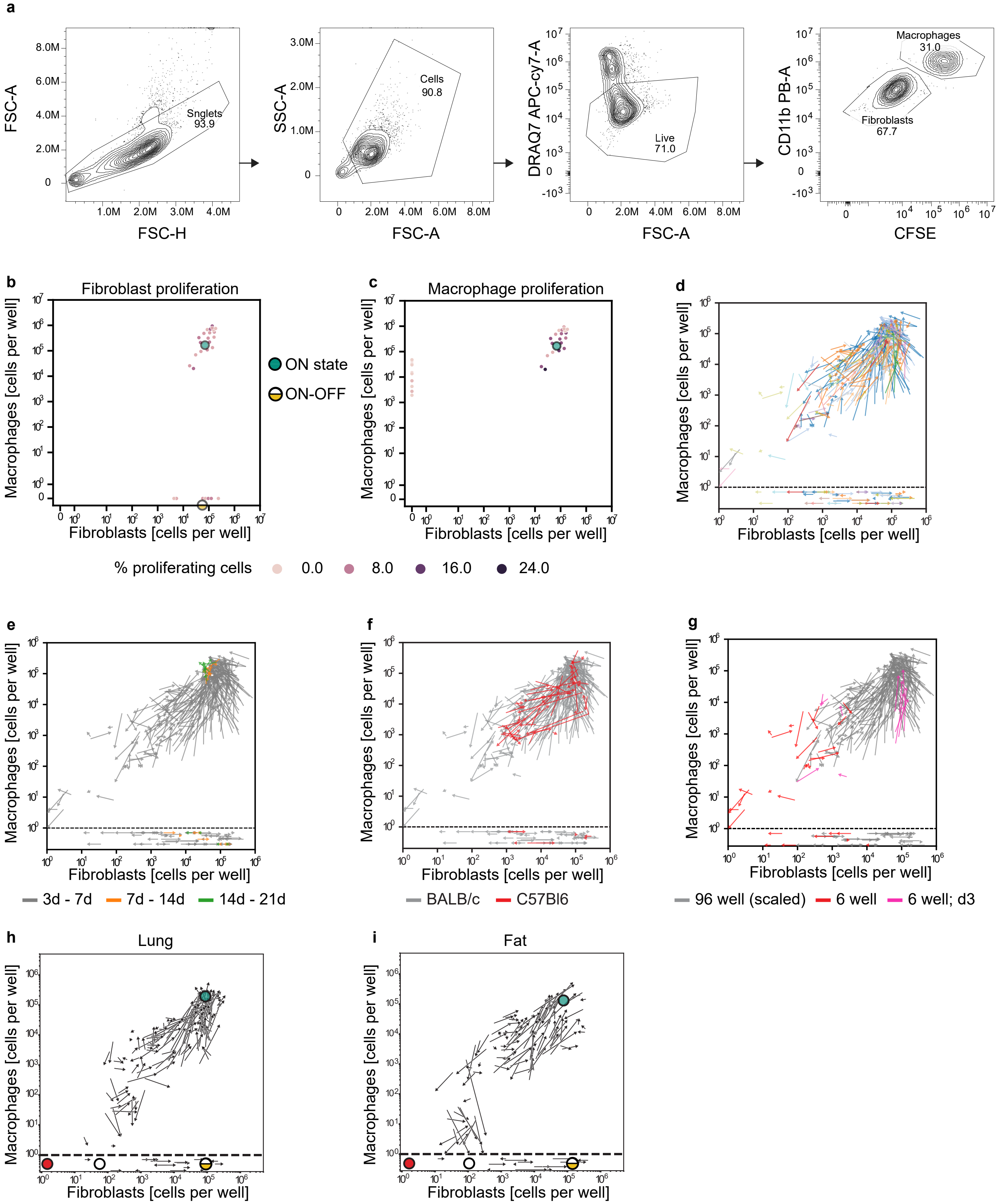

Supplementary Figure 2. The phase portrait approach is robust to biological and temporal variation. (**a**) Flow cytometry analysis strategy for the fibroblast-macrophage co-culture assay: all live single cells (DRAQ7-negative cells after debris and doublet exclusion) were analyzed. Cells staining positive for CD11b and CFSE were counted as macrophages, and cells staining negative for these markers were counted as fibroblasts. (**b-c**) Mammary fibroblasts and macrophages were co-cultured for 7 days after which EdU labeling was performed for 2h. Total fibroblast and macrophage numbers and EdU+ staining were counted by flow cytometry as described in (**a**). Total cell counts are presented as dots in the plot, and the percent of EdU+ cells is represented by shades of purple, as indicated. Data are combined from three independent experiments; n=3 biologically independent samples. (**d-g**) Tests of robustness for the phase portrait approach to measure macrophage - mammary fibroblast dynamics *In-vitro*. (**d**) Each biological replicate from the data presented in Figure 2c is presented in a different color. (**e**) An experimental phase portrait comparing dynamics at different time points - co-cultures assayed at days 7 to 14 are represented by orange arrows; co-cultures assayed at days 14 to 21 are represented by green arrows. These are overlaid on the experimental phase portrait presented in Figure 2c (gray arrows). (**f**) An experimental phase portrait comparing dynamics of cells from different mouse strains - C57BL/6 mice (n=8) are shown in red, and overlaid on the experimental phase portrait of cells from BALB/c presented in Figure 2c (gray arrows). (**g**) The arrows in Figure 2c are colored according to the following growth conditions: gray arrows represent co-cultures growing in 96 well plates, where fibroblasts and macrophages were seeded simultaneously; red arrows represent co-cultures growing in 6 well plates, where fibroblasts and macrophages were seeded simultaneously; and the pink arrows represent co-cultures growing in 6 well plates, where macrophages were added to the culture 3 days after fibroblasts were seeded. (**h-i**) Tests of robustness for the phase portrait approach using co-cultures of BMDMs with fibroblasts from either the lung (**h**) or mesometrial fat (**i**).

Mayer and Milo et al.

Supplementary Figure 3

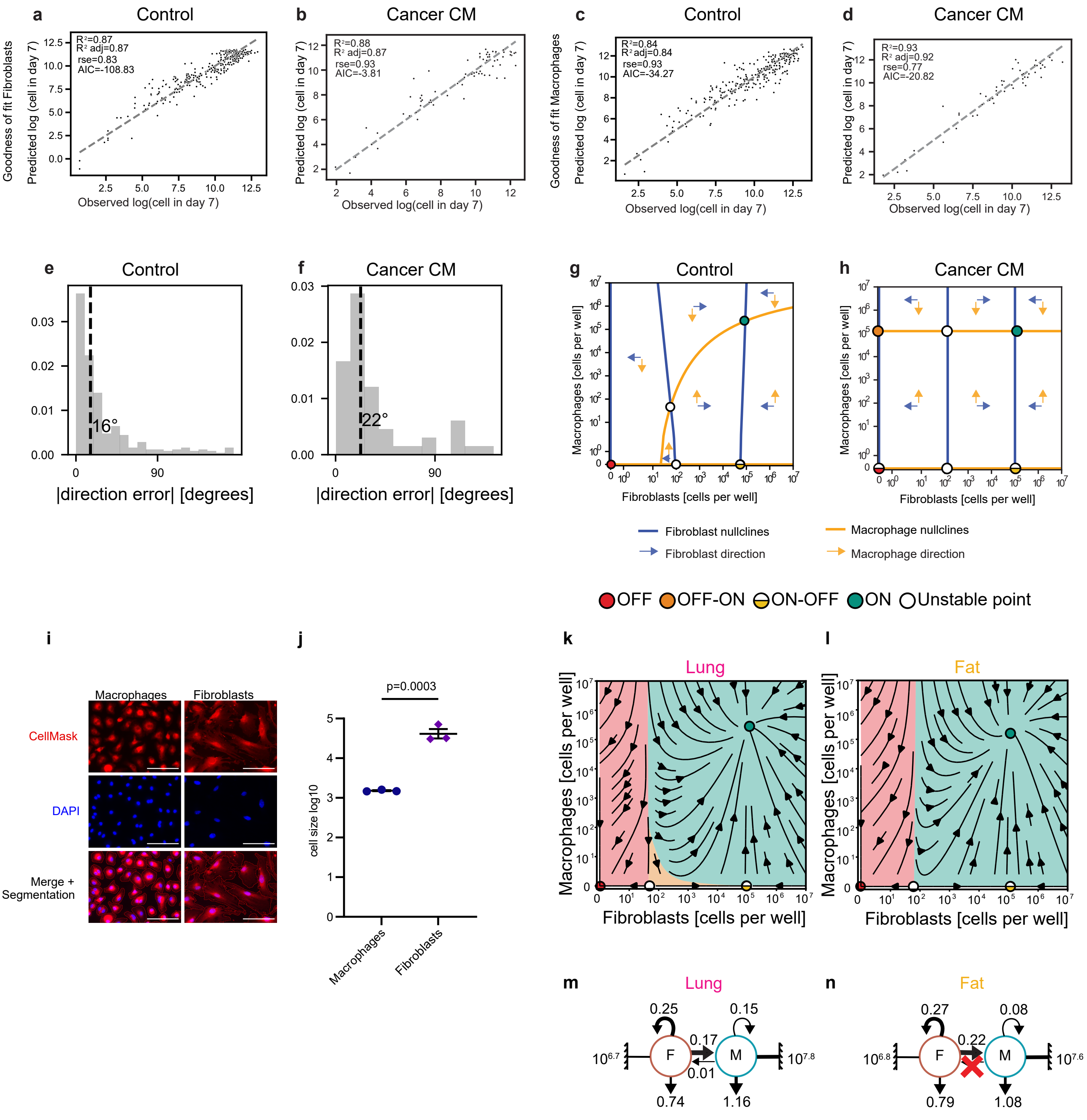

Supplementary Figure 3. Evaluation and validation of the fibroblast-macrophage circuit mathematical model. (**a-d**) Predicted cell numbers at day 7 were plotted against the observed numbers to assess the goodness of fit of the model in control medium (**a**, **c**) and in cancer CM (**b**, **d**). Dashed lines indicate perfect fit. (**e-f**) The error in prediction of the direction of growth of the cell populations: The error between the direction of the observed arrow to the direction of the predicted arrow is shown. Same source data that is used in Figure 2. (**g-h**) Nullclines of each cell population (fibroblasts in blue and macrophages in orange) for control (**g**) and cancer CM (**h**). The nullclines split the phase portrait into regions of cell population growth or decline (see arrows). The intersections between the nullclines are the fixed points of the system. (**i**) Macrophages and fibroblasts were grown in mono-culture for 7 days, fixed, and stained with CellMask to mark the plasma membrane and with DAPI to mark nuclei. Representative images are shown. Scale bar—67 μm. Image analysis was performed using Cellpose for cell segmentation (lower panels, red line) and QuPath for cell size quantification. (**j**) For each biological replicate the average cell size was calculated from 3 images. Results are shown as mean ± SEM , n=3 biologically independent samples. P-value was calculated using two -sided students' t-test. Source data are provided as a Source Data file. (**k-l**) Theoretical phase portraits showing dynamic interactions of macrophages with lung (**k**) and fat (**l**) fibroblasts (as indicated). Regions with distinct kinetics are indicated by color: in the red area cells flow to the "OFF" state (red dot); in the green area flow is to the "ON" state (green dot), and in the orange area cells flow to the "ON-OFF" state (half-yellow dot). (**m-n**) Theoretical cell circuits with the mean value of each parameter for lung (**m**) and fat (**n**).

Mayer and Milo et al.

Supplementary Figure 4

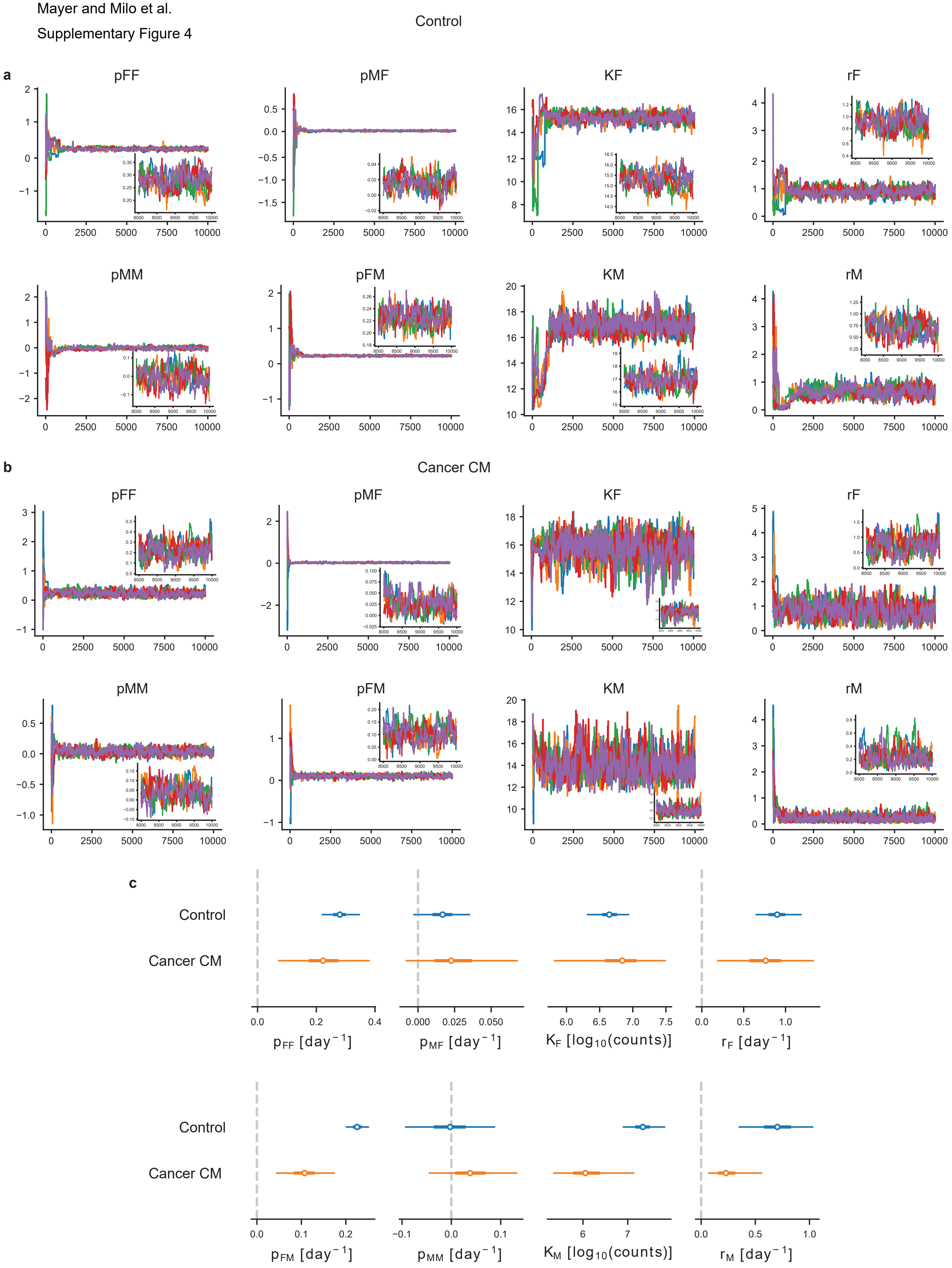

Supplementary Figure 4. Convergence and robustness of parameter calibration. (**a-b**) Validation of parameter calibration with Bayesian inference implemented in the python package PyDREAM[4]. Trace plots of five independent Markov chains for control (**a**) and cancer CM (**b**) with zoom (insets) on the last 2,000 samples to show convergence. (**c**) Parameter distributions, pooled from all 2,000 last samples of the five Markov chains. The parameter distributions are similar to the ones obtained by bootstrapping (in Figure 3c).

Supplementary Figure 5

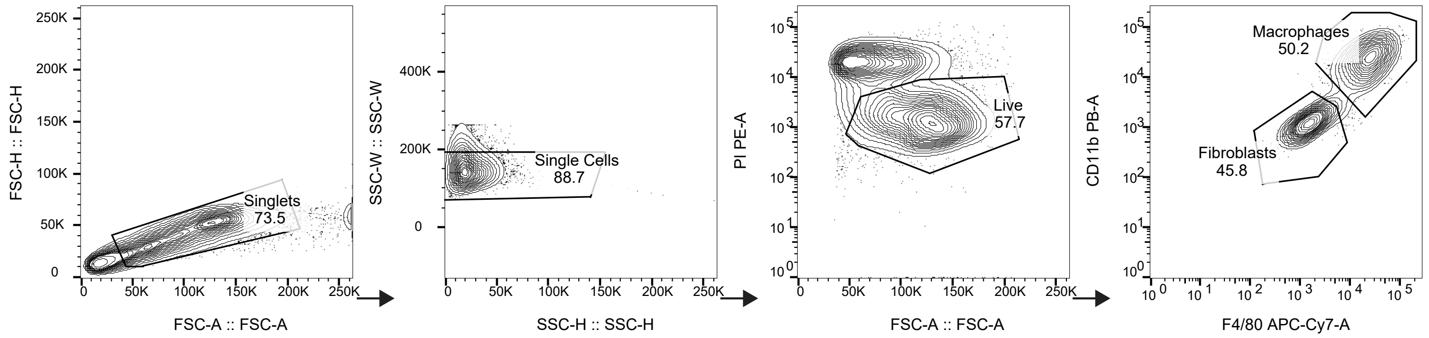

Supplementary Figure 5. FACS strategy used for RNA-seq experiments (related to Figure 4): All live single cells (PI negative cells after debris and doublet exclusion) were analyzed. Cells staining positive for CD11b-PB and F4/80 APC-Cy7 were sorted as macrophages, and cells staining negative for these markers were sorted as fibroblasts.

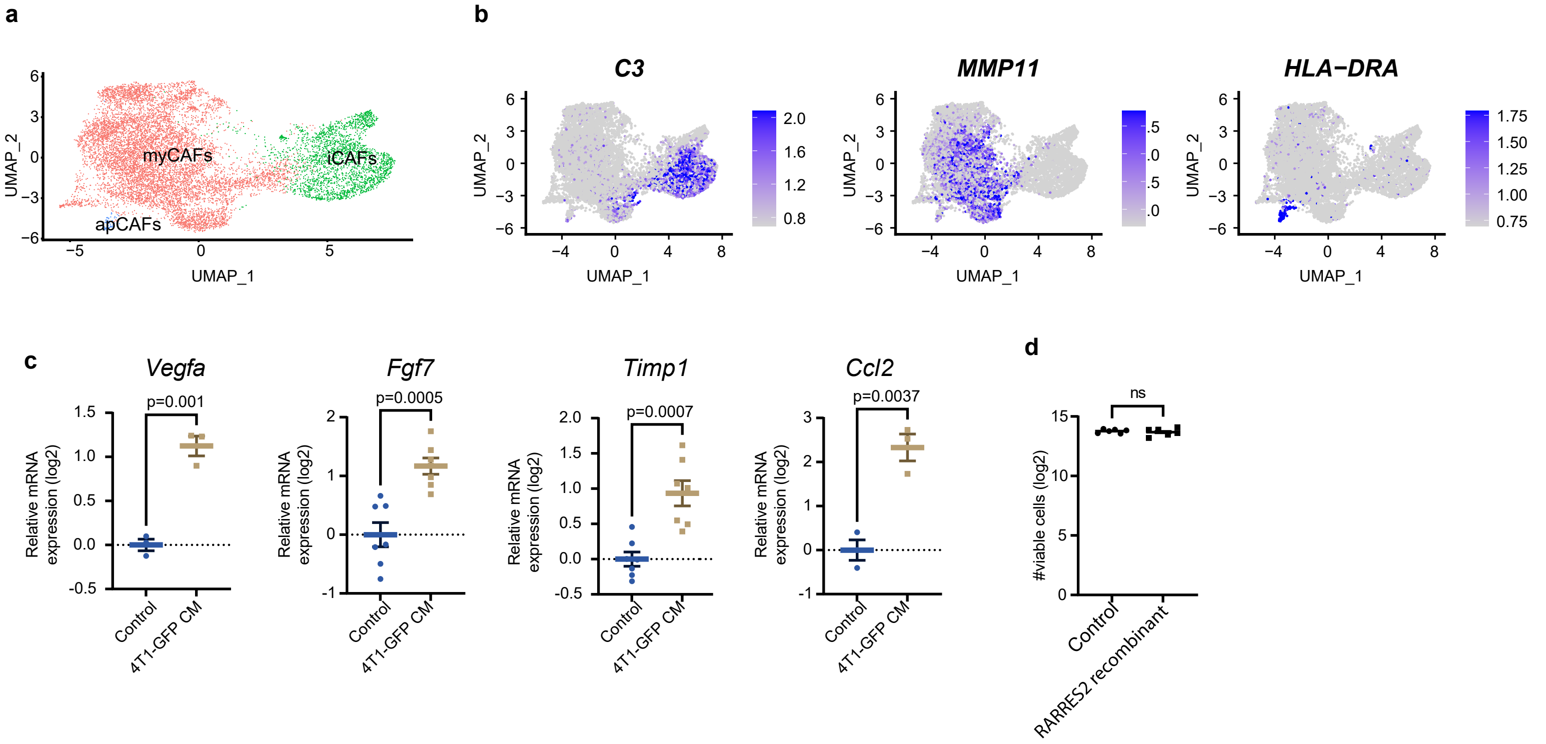

Supplementary Figure 6. The RARRES2-CMKLR1 interaction is a potential mediator of CAF-TAM signaling. (**a**) UMAP visualization of the CAF clusters in the breast TME after re-analysis of human scRNA-seq data from 32 patients [1]. (**b**) UMAP visualization shows the main markers for the CAF clusters: apCAF (*HLA-DRA*), myCAF (*MMP11*), and iCAF (*C3*). (**c**) qRT-PCR analysis of the indicated genes from fibroblasts that were mono-cultured in control or cancer CM for 72 hours. The following number of biologically independent samples was used (and is equal in both control and cancer CM): *Vegfa* (n=3), *Fgf7* (n=7), *Timp1* (n=7) and *Ccl2* (n=3). (**d**) Macrophage cell numbers were counted using a cell titer glo kit, following 24 hours of growth in mono-culture in the presence DMEM with or without 3 nM recombinant RARRES2. n=6 biologically independent samples for each condition. (**c-d**) Results are shown as mean ± SEM, P-value was calculated using two-sided students' t-test. ns marks p-values greater than 0.05. Source data are provided as a Source Data file.

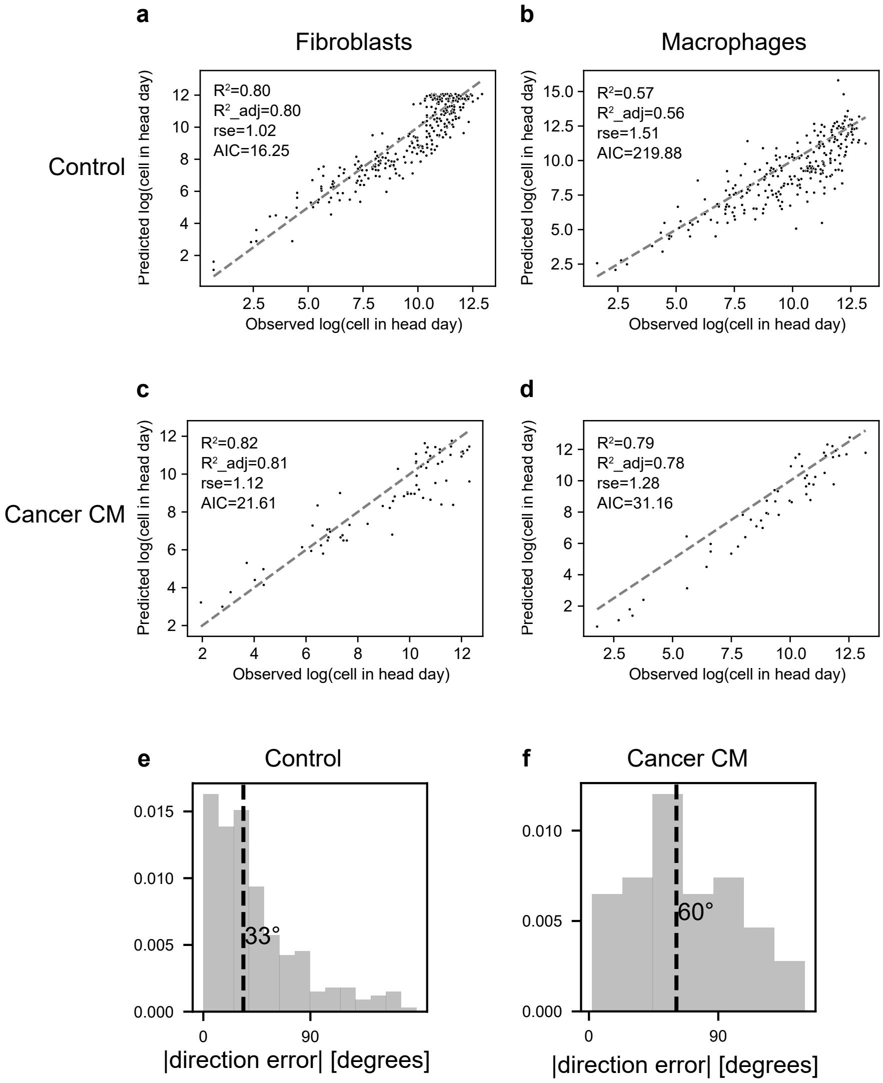

Supplementary Figure 7. Evaluation of a mathematical model with a linear dependence on cell population size, *f(X)=X* (**a-d**) The goodness of fit by plotting predicted vs. observed number of cells on day 7. (**e-f**) The error in prediction of the direction of growth of the cell populations: The error between the direction of the observed arrow to the direction of the predicted arrow is shown. Same source data that is used in Figure 2.

Supplementary Table 1. Fixed points of fibroblast-macrophage circuits.
Each pair indicates the estimated cell population numbers in the fixed points by the model and data (F,M). F - fibroblasts, M - macrophages.

	ON	ON-OFF	OFF-ON	Unstable	OFF
Control	(7.3·104, 1.9·105)	(4.2·104, 0)	-	(121, 0)	(0, 0)
				(72, 70)	
FB with 4T1 CM	(1.1·105, 1.5·105)	(6.6·104, 0)	(0, 1.5·105)	(171, 0)	-
				(45, 1.5·105)	
				(0, 0)	

Supplementary Table 2. Primer sequences

**Transcript**	**Organism**	**Forward**	**Reverse**
Hprt	mouse	CATAACCTGGTTCATCATCGC	TCCTCCTCAGACCGCTTTT
Rarres2	mouse	CCAAGAGATCGGTGTGGACAGA	CGGCTTTTTCCAGTCCTTCTTGG
Cmklr1	mouse	TTGCCGATGCACATCACCTACG	TGACAGTCAGCAGGAAGACGCT
Ccl2	mouse	GCTACAAGAGGATCACCAGCAG	GTCTGGACCCATTCCTTCTTGG
Vegfa	mouse	CTGCTGTAACGATGAAGCCCTG	GCTGTAGGAAGCTCATCTCTCC
Fgf7	mouse	GTCTACTTTCTCTTCTGGACTCC	CCAAGAGTCTCTGTTGCCTGCA
Timp1	mouse	TCTTGGTTCCCTGGCGTACTCT	GTGAGTGTCACTCTCCAGTTTGC

Supplementary Table 3. List of antibodies used in this study

Antibody	Supplier	Cat. #	clone	Dilution	Application
CD11b-Pacific blue	Biolegend	101224	M1/70	1:100	FACS
EpCAM-FITC	Miltenyi	130-117-752	REA977	1:100	FACS
CD31-FITC	Miltenyi	130-123-675	390	1:100	FACS
CD45-FITC	Miltenyi	130-110-658	REA737	1:100	FACS
Ly6C-Pacific blue	Biolegend	128014	HK1.4	1:100	FACS
PDPN-APC	Biolegend	127410	8.1.1	1:100	FACS
F4/80-APC Cy7	Biolegend	123117	BM8	1:100	FACS
CD206-BV711	Biolegend	141727	C068C2	1:100	FACS
Propidium iodide	Sigma Aldrich	P4170		1:1000	FACS
DRAQ7	Biolegend	424001		1:500	FACS
Ghost Dye Violet 450	TONBO	13-0863-T100		1:1000	FACS

References

1. A single-cell RNA expression atlas of normal, preneoplastic and tumorigenic states in the human breast. *EMBO J.* **40**, e107333 (2021).
2. Jin, S. *et al.* Inference and analysis of cell-cell communication using CellChat. *Nat. Commun.* **12**, 1088 (2021).
3. Sebastian, A. *et al.* Single-Cell Transcriptomic Analysis of Tumor-Derived Fibroblasts and Normal Tissue-Resident Fibroblasts Reveals Fibroblast Heterogeneity in Breast Cancer. *Cancers* **12**, E1307 (2020).
4. Shockley, E. M., Vrugt, J. A. & Lopez, C. F. PyDREAM: high-dimensional parameter inference for biological models in python. *Bioinformatics* **34**, 695–697 (2018).
