# Supplementary material for: The tumor microenvironment shows a hierarchy of cell-cell interactions dominated by fibroblasts
Source: Nat Commun. 2023 Sep 19;14:5810. doi: 10.1038/s41467-023-41518-w (PMC10509226; doi:10.1038/s41467-023-41518-w)
Supplement: Supplementary file 3 — Description of Additional Supplementary Files [file 41467_2023_41518_MOESM3_ESM.pdf]

Description of Additional Supplementary Files

Supplementary Data 1: Ligand receptor scores

Supplementary Data 2: Macrophages CM RNA seq

Supplementary Data 3: Fibroblasts CM RNA seq

Supplementary Data 4: CAF ssGSEA score
