# Supplementary material for: The tumor microenvironment shows a hierarchy of cell-cell interactions dominated by fibroblasts
Source: Nat Commun. 2023 Sep 19;14:5810. doi: 10.1038/s41467-023-41518-w (PMC10509226; doi:10.1038/s41467-023-41518-w)
Supplement: Supplementary file 8 — Reporting Summary [file 41467_2023_41518_MOESM8_ESM.pdf]

nature portfolio

Corresponding author(s): Ruth Scherz-Shouval

Last updated by author(s): Sep 6, 2023

Reporting Summary

Nature Portfolio wishes to improve the reproducibility of the work that we publish. This form provides structure for consistency and transparency in reporting. For further information on Nature Portfolio policies, see our Editorial Policies and the Editorial Policy Checklist.

Statistics

For all statistical analyses, confirm that the following items are present in the figure legend, table legend, main text, or Methods section.

n/a	Confirmed	
☐	☒	The exact sample size (*n*) for each experimental group/condition, given as a discrete number and unit of measurement
☐	☒	A statement on whether measurements were taken from distinct samples or whether the same sample was measured repeatedly
☐	☒	The statistical test(s) used AND whether they are one- or two-sided<br>*Only common tests should be described solely by name; describe more complex techniques in the Methods section.*
☐	☒	A description of all covariates tested
☐	☒	A description of any assumptions or corrections, such as tests of normality and adjustment for multiple comparisons
☐	☒	A full description of the statistical parameters including central tendency (e.g. means) or other basic estimates (e.g. regression coefficient) AND variation (e.g. standard deviation) or associated estimates of uncertainty (e.g. confidence intervals)
☐	☒	For null hypothesis testing, the test statistic (e.g. *F*, *t*, *r*) with confidence intervals, effect sizes, degrees of freedom and *P* value noted<br>*Give P values as exact values whenever suitable.*
☐	☒	For Bayesian analysis, information on the choice of priors and Markov chain Monte Carlo settings
☐	☒	For hierarchical and complex designs, identification of the appropriate level for tests and full reporting of outcomes
☐	☒	Estimates of effect sizes (e.g. Cohen's *d*, Pearson's *r*), indicating how they were calculated

Our web collection on statistics for biologists contains articles on many of the points above.

Software and code

Policy information about availability of computer code

Data collection

FACS analysis was performed using CytoFlex-S (Beckman Coulter) with CytExpert software .
FACS-sorting was performed using FACSMelody instrument (BD-biosciences) with BD FACSChorus software.
Imaging data was collected using a Nikon Eclipse Ci microscope with a ×10 objective.
Illumina NextSeq 500 was used for RNA-sequencing.
Sirius Red collagen staining was measured by Cytation 5-Imaging Reader (Biotek).
Chemerin/RARRES2 kit following themanufacturer's instructions (Boster, EK1330).
Macrophage count using Glo lysis buffer (Promega, E2661) according to the manufacturer's instructions. Luminescence was measured in relative light units using a plate reader.

Data analysis

Image analysis was performed by QuPath (v.0.3.2).
FACS analysis was performed using flowjo software v.10.7.1.
For the ligand-receptor analysis we used the CellChat and NicheNet R packages (https://github.com/sqjin/CellChat ; https://github.com/saeyslab/nichenetr).
Statistical analysis and visualization were performed using R (Versions 3.6.0 and 4.2.0, R Foundation for Statistical Computing Vienna, Austria) and Prism 9.1.1 (Graphpad, USA).
Pathway analysis was performed using Metascape. We used the Seurat v.4.0.0 method in R v.4.2.2 to reanalyze published scRNA-seq data.
Phase portraits and parameter inference of the cell circuits were calculated using Python and scipy package. Data analysis scripts are available in https://github.com/tomermilo/fibroblast_macrophage_circuits/tree/master
Mathematica 13.3 was used for the network motif analysis. Phase portraits and parameter inference of the cell circuits were calculated using

Python 3.7.4 and scipy package 1.7.3.

For manuscripts utilizing custom algorithms or software that are central to the research but not yet described in published literature, software must be made available to editors and reviewers. We strongly encourage code deposition in a community repository (e.g. GitHub). See the Nature Portfolio guidelines for submitting code & software for further information.

Data

Policy information about availability of data

All manuscripts must include a data availability statement. This statement should provide the following information, where applicable:

- Accession codes, unique identifiers, or web links for publicly available datasets
- A description of any restrictions on data availability
- For clinical datasets or third party data, please ensure that the statement adheres to our policy

All RNA-seq data from this study was deposited in the in the Gene Expression Omnibus (GEO) :GSE217737. Scripts and data needed to reconstruct the phase portrait analysis and figures are available https://github.com/tomermilo/fibroblast_macrophage_circuits/tree/master
The breast cancer scRNA-seq datasets of human and mouse, which were used in this study, are available on the as GEO: GSE161529 and https://datadryad.org/stash/dataset/doi:10.6071/M3238R. Bulk RNA-seq data that support the findings of this study were deposited in GEO: GSE218197. The gene signatures of protumorigenic TAMs and in-vivo CAFs can be accessed via GEO: GSE195858 and GSE195865. The source data are provided with this paper.
All other data supporting the findings of this study are available from the corresponding author on reasonable request.

Research involving human participants, their data, or biological material

Policy information about studies with human participants or human data. See also policy information about sex, gender (identity/presentation), and sexual orientation and race, ethnicity and racism.

Reporting on sex and gender	Female BALB/c and C57BL/6 mice were used since the focus of this study is breast cancer, occurring mostly in females
Reporting on race, ethnicity, or other socially relevant groupings	The scRNA-seq data used in this study was obtained from a published breast cancer study (Pal et al., EMBO, 2023). The published study collected mostly samples from female patients, and only 2 samples from male patients. Due to the very limited number of male patients we excluded these patients from our analysis.
Population characteristics	*Describe the covariate-relevant population characteristics of the human research participants (e.g. age, genotypic information, past and current diagnosis and treatment categories). If you filled out the behavioural & social sciences study design questions and have nothing to add here, write "See above."*
Recruitment	*Describe how participants were recruited. Outline any potential self-selection bias or other biases that may be present and how these are likely to impact results.*
Ethics oversight	IACUC protocol #05420621-2

Note that full information on the approval of the study protocol must also be provided in the manuscript.

Field-specific reporting

Please select the one below that is the best fit for your research. If you are not sure, read the appropriate sections before making your selection.

☒ Life sciences ☐ Behavioural & social sciences ☐ Ecological, evolutionary & environmental sciences

For a reference copy of the document with all sections, see nature.com/documents/nr-reporting-summary-flat.pdf

Life sciences study design

All studies must disclose on these points even when the disclosure is negative.

Sample size	-Organ co-culture experiments were performed at least 3 times and the results are combined from the following total number of biological replicates: macrophages only: n=5; mammary: n=24; lung: n=16; fat: n=10 mice.<br>-4T1 CM co-culture experiments are combined from 5 independent experiments (performed in parallel to control media co-cultures); n=12 mice for the cancer CM and n=6 for control medium.<br>-Macrophage count in the presence of normal epithelial and cancer CM: Data are combined from three independent experiments, with n=8 biological replicates.<br>-Edu staining: n=3 mice, was repeated in 3 independent experiments .<br>-Co-culture experiments from C57BL/6 mice were performed at least 3 times and the results are combined from the following total number of biological replicates: n=8.<br>For the in-vitro phase portrait experiments, the sample sizes were chosen based on previous study that used the phase portrait approach (Zhou et al.,2018, Cell).<br>-Sirius Red staining was repeated in 3 independent experiments with the following total of biological replicates: Macrophages only: n=4; mammary: n=3. For this experiment, the sample sizes were chosen based on a previous study that utilized this staining method (Levi-Galibov et al., 2020, Nat Comm.)<br>-Bulk RNA-seq was performed from the following number of biological replicates: macrophages in control n=3, macrophages in cancer CM:

	n=4, fibroblasts in control n=3, Fibroblasts mono-cultures in cancer CM: n=2, Fibroblasts co-cultures in cancer CM: n=4mice.<br>-For cell size images: macrophages only: n=3; fibroblasts n=3, was repeated in 3 independent experiments.<br>- Macrophages Flow cytometry analysis was of marker CD206: n=9 biological replicates from a total of 3 separate experiments.<br>-qPCR of Rarres2 and Cmklr1 : Data are combined from at least three independent experiments, for Rarres2: Fibroblasts mono-cultured in control or in cancer CM n=5, fibroblasts co-cultured in control n=5 or in cancer CM n=4 biological replicates. For Cmklr1: Macrophages mono-cultured in control n=4 or in cancer CM n=9, macrophages co-cultured in control or in cancer CM n=7 biological replicates.<br>- Elisa for RARRES2: Data are combined from at least three independent experiments, 4T1=4; CAF=5 biological replicates.<br>-Transwell migration assay:Data are combined from at least three independent experiments, with macrophages in the presence of control medium n=10, or 4T1 cancer CM with n=9 or without n=12recombinant RARRES2.<br>For Bulk RNA-seq and the in-vitro experiments, the sample sizes were chosen based on a previous study that used these methods (Friedman et al., 2020, Nat Cancer).
Data exclusions	In the Bulk RNA-seq, two libraries (two samples of mono-cultured mammary fibroblast) were excluded due to technical problems with sequencing, as no reads were detected. In Figure 5g, i-j, one patient exhibited values in RARRES2 expression that exceeded 2 standard deviations of their group mean. As this exceeded the predetermined criterion of 2 standard deviations, the patient was defined as an outlier and subsequently excluded from the analysis of the mentioned figures.
Replication	All replication attempts were successful. All the in vitro experiments are combined from at least three independent experiments, each with 2-4 independent mice.
Randomization	All mice were purchased and housed together at the same age and tissues/tumors harvested from them were randomly assigned into experimental groups.
Blinding	All experiments did not necessitate blinding since the analyses were performed using computational algorithms and not human assessment.

Reporting for specific materials, systems and methods

We require information from authors about some types of materials, experimental systems and methods used in many studies. Here, indicate whether each material, system or method listed is relevant to your study. If you are not sure if a list item applies to your research, read the appropriate section before selecting a response.

Materials & experimental systems

n/a	Involved in the study
☐	☒ Antibodies
☐	☒ Eukaryotic cell lines
☒	☐ Palaeontology and archaeology
☐	☒ Animals and other organisms
☒	☐ Clinical data
☒	☐ Dual use research of concern
☒	☐ Plants

Methods

n/a	Involved in the study
☒	☐ ChIP-seq
☐	☒ Flow cytometry
☒	☐ MRI-based neuroimaging

Antibodies

Antibodies used	CD11b-Pacific blue, Biolegend , cat: 101224 ,clone: M1/70, dilution: 1:100, used for FACS<br>EpCAM-FITC, Miltenyi , cat: 130-117-752 ,clone: REA977 , dilution: 1:100, used for FACS<br>CD31-FITC, Miltenyi , cat: 130-123-675 ,clone: 390 , dilution: 1:100, used for FACS<br>CD45-FITC, Miltenyi , cat: 130-110-658 ,clone: REA737 , dilution: 1:100 ,used for FACS<br>Ly6C-Pacific blue , Biolegend , cat: 128014 ,clone: HK1.4 , dilution: 1:100, used for FACS<br>PDPN-APC , Biolegend , cat: 127410 ,clone: 8.1.1 , dilution: 1:100 ,used for FACS<br>F4/80-APC Cy7, Biolegend , cat: 123117 ,clone: BM8 1, dilution: :100 , used for FACS<br>CD206-BV711 , Biolegend , cat: 141727 ,clone: C068C2 , dilution: 1:100 , used for FACS<br>Propidium iodide , Sigma Aldrich , cat: P4170 , dilution: 1:1000, used for FACS<br>DRAQ7 , Biolegend , cat: 424001 , dilution: 1:500, used for FACS<br>Ghost Dye Violet 450, TONBO , cat: 13-0863-T100 , dilution: 1:1000, used for FACS
Validation	All antibodies were tested by the manufacturer for the relevant applications to ensure specific staining to the antigen without cross-reactivity. The antibodies for FACS were utilized as used by us in (Friedman et al., 2020, Nat Cancer), (Levi-Galibov et al., 2020, Nat Commun), and (Shaashua et al., 2022, Nat Commun).<br>In addition, all antibodies used for flow cytometry were calibrated on live mouse cells to ensure specific staining prior to use for FACS analyses and sorting.

Eukaryotic cell lines

Policy information about cell lines and Sex and Gender in Research

Cell line source(s)	4T1 female mouse mammary carcinoma cells were kindly provided by Dr. Zvi Granot, Hebrew University of Jerusalem. These cells were transduced to express green fluorescent protein (GFP) using the FUW-GFP vector.
Authentication	4T1 line was purchased by Zvi Granot from the ATCC and we profiled the cells by RNA-sequencing .
Mycoplasma contamination	All cell cultures were tested negative for mycoplasma contamination.
Commonly misidentified lines (See ICLAC register)	No commonly misidentified lines were used .

Animals and other research organisms

Policy information about studies involving animals; ARRIVE guidelines recommended for reporting animal research, and Sex and Gender in Research

Laboratory animals	Wild-type BALB/c and C57BL/6 female mice at the age of 8 weeks. The light-dark cycle was 12 hours. The ambient temperature was 22 degrees Celsius and humidity was between 35 to 55%.
Wild animals	The study did not involve wild animals.
Reporting on sex	We were using only female BALB/c and C57BL/6 mice because we are investigating breast cancer in women, and this is the parallel model.
Field-collected samples	The study did not involve samples collected from the field.
Ethics oversight	All animal studies were conducted in accordance with the regulations formulated by the Institutional Animal Care and Use Committee (IACUC; protocol #05420621-2) from the Weizmann Institute of Science (WIS).

Note that full information on the approval of the study protocol must also be provided in the manuscript.

Flow Cytometry

Plots

Confirm that:

- [x] The axis labels state the marker and fluorochrome used (e.g. CD4-FITC).
- [x] The axis scales are clearly visible. Include numbers along axes only for bottom left plot of group (a 'group' is an analysis of identical markers).
- [x] All plots are contour plots with outliers or pseudocolor plots.
- [x] A numerical value for number of cells or percentage (with statistics) is provided.

Methodology

Sample preparation	Fibroblasts and macrophages were harvested from tissue culture plates by incubation with a non-enzymatic cell dissociation solution, washed, and transferred to round-bottom 96-well plates.
Instrument	CytoFlex-S (Beckman Coulter) was used for cell counting, and FACS sorting was preformed using FACSMelody instrument (BD-biosciences).
Software	CytExpert is the for CytoFlex-S software, BD FACSChorus is the for FACSMelody instrument (BD-biosciences) software.
Cell population abundance	The abundance of the relevant cell populations are mentioned in Figure S2A and S6A
Gating strategy	Sorting strategy: All live single cells (PI negative cells after debris and doublet exclusion) were sorted. Cells staining positive for anti-CD11b-Pacific blue and anti-F4/80-APC Cy7 were sorted as macrophages, and cells staining negative for these markers were sorted as fibroblasts.<br>FACS analyzer: Dead cells were excluded using DRAQ7. The cells were then counted by flow cytometry using CFSE and anti-CD11b-Pacific blue antibody as positive markers for macrophages. Cells stained negatively for these markers were counted as fibroblasts.

- [x] Tick this box to confirm that a figure exemplifying the gating strategy is provided in the Supplementary Information.
